# Ethical challenges and opportunities in the development and approval of novel therapeutics for rare diseases

**DOI:** 10.1177/27550834231177507

**Published:** 2023-06-06

**Authors:** Djurdja Djordjevic, Andrew McFadyen, James A Anderson

**Affiliations:** 1 The Hospital for Sick Children, Toronto, ON, Canada; 2 The Isaac Foundation, Campbellford, ON, Canada

**Keywords:** Drug approval, rare diseases, ethics research, data collection, Canada, resource allocation

## Abstract

The development of novel therapeutics for rare “orphan” diseases has brought a growing tension between the desire to accelerate access to these breakthrough therapies and the need to generate quality evidence regarding their safety and efficacy. Accelerating the pace of drug development and approval may facilitate the rapid delivery of benefits to patients and cost savings for research and development, which theoretically improves affordability of drugs for the health system. However, several ethical challenges arise with expedited approval, compassionate release of drugs, and subsequent study of drugs in “real-world” settings. In this article, we explore the changing landscape of drug approval and the ethical challenges expedited approval creates for patients, caregivers, clinicians, and institutions, and propose tangible strategies to maximize the benefits of “real-world” data acquisition while mitigating risks to patients, clinicians, and institutions.

## Introduction

The development of novel therapeutics for rare “orphan” diseases has brought a growing tension between the desire to accelerate access to these breakthrough therapies, and the need to generate quality evidence regarding their safety and efficacy. Accelerating the pace of drug development and approval may facilitate the rapid delivery of benefits to patients and cost savings for research and development, which theoretically improves affordability of drugs for the health system. However, several ethical challenges arise with accelerated approval, compassionate release of drugs, and subsequent study of drugs in “real-world” settings. In this article, we explore the changing landscape of drug approval and propose strategies to maximize the benefits of “real-world” data acquisition while mitigating risks to patients, clinicians, and institutions.

## Drug development, approval, reimbursement, and access

Regulatory processes for drug development, approval, reimbursement, and access are now well established nationally and internationally. These stringent regulatory processes offer important legal and ethical protections for human participants in research, as well as for patients and consumers of these products. However, these processes have also resulted in a trade-off with efficiency, prompting decades of criticism regarding drug research and development processes. To address concerns, regulatory processes in many jurisdictions have undergone numerous revisions. Notably, processes for accelerated drug approval and compassionate access have been developed to expedite access to particular lifesaving novel therapeutics.^1–4^

Although our aim in this article is to highlight challenges to, and make recommendations for, many health systems, for the purposes of this discussion, it will be helpful to work with a specific, more detailed example. Since the authors live and work in Canada, we will use the Canadian system for this purpose.

In Canada, priority review allows faster approval of sufficiently effective drugs for the treatment of conditions which are life-limiting or very severe, or where there are no other drug options.^5^ Through this mechanism, a review takes 180 days instead of 345 days for other new drugs. Conditional market approval of a drug can also be achieved by Health Canada through a Notice of Conditions (NOC/c), a policy allowing for faster market access to drugs deemed lifesaving in clinical trials.^6^ According to Health Canada, a drug eligible for NOC/c must have “substantial evidence of clinical effectiveness” in which two or more “well controlled clinical studies” show the drug is effective as assessed by scientific experts, or in special circumstances just one large trial with additional evidence. “Promising” clinical evidence such as Phase II studies or other markers of drug efficacy are also considered within the NOC/c policy to determine eligibility for accelerated approval.^6^

Once a drug is approved by Health Canada, it undergoes a health technology assessment (HTA) by the Canadian Drug and Health Technology Agency (CADTH). CADTH reviews new drug approvals and provides reimbursement recommendations to Provinces and Territories. If a positive recommendation is rendered, reimbursement negotiations can begin with the pan-Canadian Pharmaceutical Alliance (pCPA). The pCPA’s mandate is to negotiate a price for drugs on behalf of most jurisdictions in Canada. These additional review mechanisms can be lengthy, leading to delays in access for patients, with the median length of time from Health Canada approval to completion of CADTH review and pCPA reimbursement negotiations being 21 months.^7^ These reviews often recommend additional criteria for coverage, further limiting access for patients, and almost universally stipulate that a substantial price reduction must be realized prior to being listed on drug formularies.^7^

Post market approval, clinicians may apply to the jurisdiction’s respective Exceptional Access Program for reimbursement of high-cost drugs approved for specific circumstances. The patient must meet specific inclusion and exclusion criteria to receive funding, and for those outside of these criteria, there may be exceptions made. Alternatively, patients may undertake other methods for obtaining private funding for the medication.

For non-marketed drugs, clinicians can apply to Canada’s Special Access Program (SAP). This is available for treatment of patients with serious or life-threatening conditions when other therapies have failed or are unavailable (C.08.010 and C.08.011 of the *Food and Drug Regulations*). The SAP authorizes temporary use of unapproved drugs that cannot otherwise be distributed in Canada. These are generally one-off requests and rarely provide sufﬁcient data to the manufacturer to build a full New Drug Submission application. However, it provides a much-needed access mechanism for select patients.^8^

Although the details reviewed above are particular to Canada, as noted previously, similar programs exist in the United States, the United Kingdom, the European Union (EU), and other jurisdictions. Furthermore, these programs have been developed in response to similar challenges. For example, many publicly funded drug programs incorporate similar restrictive formularies to help control costs and limit the burden on health care resources associated with expensive drugs.^9^ Many of these same countries rely on a bespoke HTA review body to help inform reimbursement decisions,^10^ much like Canada does, and similarly incorporate a reliance on cost-effectiveness models such as QALYs to help inform reimbursement decisions.^9^

Furthermore, while Canada is a publicly funded system, it is considered a “decentralized,” pluralistic health system because health services are funded at a national level, but coverage decisions are made at a Provincial level.^11^ This results in a disparity of reimbursed access and potentially differing inclusion-criteria for some expensive drugs across the country,^12^ and similar challenges exist in other decentralized systems, including Spain and Italy.^11^ Moreover, these challenges can be seen in other pluralistic health systems, like the United States, where health insurers may differ on whether expensive novel therapeutics ought to be reimbursed, and for whom, after conducting their own, separate HTA reviews.^11^

Finally, as noted above, many countries have similar accelerated approval pathways, designed to ensure faster access to drugs compared with historical regulatory processes. For instance, Canada’s priority review process is similar to the Innovative Licensing and Access Pathway in the United Kingdom,^2^ the accelerated approval pathway in the United States,^4^ and the accelerated assessment process in the EU.^3^

### Challenges with standard regulatory processes

Historically, regulatory approval processes, like those of the Food and Drug Administration (FDA) and Health Canada, proceed stepwise through trial phases 1 to 3, culminating in large, expensive, preferably randomized clinical trials (RCTs). Small patient populations, coupled with the heterogeneous nature of many rare diseases, create significant challenges for the conduct of robust trials in the rare disease space.^13^ In addition, the natural history of many rare diseases is not well understood, impacting researchers’ ability to design appropriate trials and identify clinically meaningful endpoints in their trial design.^13^ Thus, when trials are conducted in this space, concerns about internal and external validity are typical. And when drugs are approved, it is often without adequate evidence in many patient groups, making extrapolation of data concerning risks and benefits to different clinical settings and patient populations very difficult.^14^

For these and other reasons, standard regulatory processes have drawn the ire of players at every step of the drug development pipeline. Industry has long raised concerns about the length and cost of the development and approval process. Funders worry that calculating cost-effectiveness is challenging for some novel therapeutics due to differences between trial patients and those seeking treatment in the field.^15^ Clinicians raise parallel concerns about the absence of meaningful data for clinical decision-making. And patients, families, and patient advocates argue that regulations are too stringent and delay access to lifesaving drugs.

Some of these tensions are exacerbated by the current landscape of novel therapeutics for rare diseases. With improvement in our drug development technologies and the advent of precision therapy, drugs are becoming more targeted in terms of their mechanism of action, and more selective in terms of their target population.^16^ The idea that these drugs are likelier to be more effective, as evidenced by their lower clinical trial failure rate,^17^ has prompted pressure both from industry for faster approval and from patients and patient advocates for faster access. This is particularly true for therapies that have been developed for rare diseases without previously available treatments.

### Challenges with accelerated approval

While accelerated approval processes do address some of the challenges associated with standard regulatory processes, they have also created other challenges. In this section, we consider several associated ethical challenges for patients, clinicians, institutions, and society more broadly. Careful consideration of these challenges is critical to developing appropriate strategies to mitigate them.

#### Ethical challenges for patients and caregivers

In large part, the success of the orphan drug market turns on the lack of other treatment options for many rare diseases.^18^ This is a striking advantage to the pharmaceutical companies developing a unique drug, whose targeted marketing is especially effective with vulnerable populations. Autonomous decision-making is problematic in this context, as pursuing access to an experimental therapy may be the only “choice” available. Patients and caregivers are likely to overestimate benefit and minimize risk. For patients and caregivers anxious for expeditious access to a promising experimental agent, a rapid path through clinical validation will often result in many unanswered questions. Indeed, given the limited evidence concerning the true risks and benefits of many drugs obtained via special access or post-accelerated approval, it is unclear whether an individual patient or caregiver can truly provide informed consent.

Early access to novel medications outside of the standard regulatory process also raises concerns about equity. Inequities related to clinical trial participation are widespread and well documented.^19–21^ Access via an Expanded Access Program (EAP) or SAP exacerbates these concerns,^22^ and often, only a motivated, informed, and well-connected subset of the patient population will achieve access through these programs. When access requires private pay or travel out of province or country, equity concerns are exacerbated even further.

Accelerated approval also raises equity concerns as limited data tend to result in narrower criteria for reimbursement and/or variable reimbursement across funding jurisdictions. Consider, for example, recent experience with Zolgensma in Canada. Health Canada approved Zolgensma for use in children 2 years or younger with infantile onset Spinal Muscular Atrophy (SMA) or those with 3 or fewer copies of *SMN2.* A subsequent review by CADTH recommended narrowing eligibility to patients under 6 months of age, citing a lack of efficacy data for patients older than 6 months.^23^ For patients and caregivers anxious for expeditious access whose children were older than 6 months, Zolgensma’s rapid path through clinical trials resulted in many unanswered questions. Currently, access for many patients remains severely limited.

#### Ethical challenges for clinicians and institutions

Accelerating drug approval provides less opportunity for testing the parameters of safe and effective use, which also means that the assumptions about targets, dosage, and safety are not as thoroughly tested.^24^ While the high-stakes nature of many of the diseases may arguably justify a higher threshold of acceptable adverse effects, this can be problematic.

For the clinician, weighing a treatment’s possible benefits against potential for harm for an individual patient is often difficult; this challenge is exacerbated when there are limited data available for decision-making. Clinicians, too, are torn between evidence-based practice and access. Tension is ratcheted up by additive pressure from the media, institutions, and families themselves. The precedent set by a clinician and institution is also critical to consider, as more patients and families will desire access to these drugs once the door is open.

There are also important implications for oversight. For the clinician and institution, there is inherent risk in administering an accelerated access or off-market drug. In our Canadian institutions, SAP is applied for directly by the clinician to the company. Without an independent institutional review, perceived or actual conflicts of interest—on the part of the company, institution, and/or clinicians—may undermine trust in the process. While the drug may improve or prolong the life of a patient, the institution will also bear some burden of responsibility should there be adverse events or additional medical and other supports required by the patient.

The SAP agreements themselves also dictate liability and risk allocation, seeking to place risk and responsibility on clinicians and hospitals while the suppliers are protected from legal exposure. Post licensure, drug companies may not be incentivized to undertake work to continue demonstration of safety and efficacy. This responsibility then falls on institutions and care providers who administer and monitor these treatments without adequate information regarding outcome measures. With the cost-effectiveness that results from acceleration of development and approval, pharmaceutical companies ultimately benefit from this expanded use while offloading the costs and, importantly, many of the risks associated with overseeing the distribution of these drugs.

#### Broader ethical implications

Finally, there are a number of far-reaching clinical and societal implications regarding how we conduct research on novel therapeutics. As more high-cost therapies are developed for orphan diseases, early access and accelerated approval may become the norm rather than the exception. Developed to speed access to breakthrough therapeutics, early access programs are also effective marketing tools through which manufacturers can leverage patient groups and media sources to push for approval.

What begins as an ostensibly altruistic mechanism for distributing drugs to patients in need may become a strategy for distributing many, potentially undermining the regulatory process, shifting costs and risk from companies to the public, and slowing the development of clinical knowledge. From a financial standpoint, there will be an increasingly high burden on the public health care system to fund and sustain these expensive therapeutics without first having robust evidence of their efficacy in specific patient groups. Ultimately, these concerns highlight the critical need to find a method for balancing efficiency with acquisition of safety and efficacy data in a rigorous and ethically sound manner.

Finally, the equity concerns faced by patients and families noted previously become societal concerns at scale. Consider recent experience with two new drugs developed for Alzheimer’s disease (which is far from rare): Aduhelm and Leqembi. These drugs were approved through the FDA’s accelerated approval pathway in early 2023. Accelerated approval is meant to ensure access is provided to patients without other options, but for many patients with Alzheimer’s hoping to access Aduhelm and Leqembi, this has not happened. Shortly after the accelerated approval of Aduhelm in 2021 and Leqembi in 2022, the Centers for Medicare and Medicaid Services (CMS) declined to provide reimbursement for patients unless they met very specific, and narrow, inclusion criteria.^25,26^ A subset of patients gained access, many did not. In addition, while the FDA approved Aduhelm, many other countries, Canada and the United Kingdom included, declined to approve the drug, dashing hope for patients and their caregivers. In the rare context, the same scenario played out after the FDA granted accelerated approval to Serepta’s drug Exondys 51, designed to treat Duchenne Muscular Dystrophy. While patients and patient organizations anticipated rapid access post-approval, insurers refused to pay for the drug, citing lack of efficacy evidence as their rationale.^27^ Again, due to the inherent limitations of the accelerated approval process in terms of the breadth and depth of the data produced, these outcomes were predictable.

## Overview of issues and strategies for change

### Call for change: balancing accelerated approval with real-world data collection

Given the unique nature of orphan diseases, approval and reimbursement of orphan drugs using a different framework and a streamlined process are necessary to allow for improved access. Importantly, early availability of promising drugs outside of clinical trials (and their narrow inclusion criteria) through EAPs and SAPs *does* improve access to potentially lifesaving treatments. Due to the low prevalence of rare diseases, however, early access and/or approval virtually guarantees that drugs will be accessed or approved before data can be collected on a variety of sub populations suffering from the disease.

N-of-1 treatments for rare diseases raise a similar but different problem. Looking toward the future, we anticipate an increasing number of individualized therapies designed for a small subset, or even singular patients with a particular genetic variant. This process is exemplified by gene therapies recently administered to and developed for singular patients.^28,29^ The N-of-1 process is almost always characterized by urgency and, thus, parallel concerns about minimal data for decision-making. Here, again, we see the characteristic tension between the desire to accelerate access to these breakthrough therapies, and the need to generate quality evidence regarding their safety and efficacy.

To maximize the benefits of accelerated access and balance, the sometimes considerable risks involved, subsequent follow-up, and data collection are ethically required. EAP and SAP programs provide an opportunity to study novel drugs in real-world populations, potentially facilitating more robust research on drug safety and efficacy in a greater variety of populations than within clinical trials.

Significant challenges associated with real-world data (RWD) acquisition remain, however, including the current absence of adequate frameworks and infrastructure for the responsible collection of RWD. A balance must therefore be found to complement tight clinical trials with loose pragmatic, real-world evidence (RWE) and data collection. Striking such a balance will require regulatory changes to create a sustainable trajectory for emerging therapeutics. In part, standardizing the data acquisition and study of drugs post market approval would help mitigate the risks associated with the trade-off between methodological rigor and access outside of clinical trials in this context. The risks associated with these changes should be manageable provided we are deliberate about what deviations from standard protocols are acceptable and are mindful of the ethical challenges involved.

### Proposed strategies to improve the Canadian orphan drug framework

We recommend the following strategies be included in a Canadian orphan drug framework. Thought these recommendations speak to specific features of the Canadian system, we believe analogous strategies would be useful in other jurisdictions:

First, pharmaceutical companies should be bound to undergo a triple-track review process if their drug is granted priority review status with Health Canada. Mandatory triple-track submission would ensure reviews by Health Canada and CADTH, and interim negotiations with pCPA take place concurrently, reducing the median time from drug approval to listing on provincial formularies. Priority review indicates a new drug may be lifesaving or treat a patient population with high unmet needs, and all stakeholders have a duty to ensure a fast-track process leads to accelerated access for patients. A triple-track review ought to alleviate delays seen in the consecutive processes.

Second, we call on CADTH to expedite and publish guidance for use of RWE and RWD to complement clinical trial evidence used to evaluate the efficacy of orphan drugs in rare disease patient populations. The vast majority of rare disease drugs receiving a positive recommendation from CADTH have stringent access criteria placed on their use, largely due to limited efficacy data within heterogeneous disease groups. Such criteria limit access to a very small subset of the patient population approved by Health Canada, leaving many patients without potentially lifesaving medications. This guidance could help manufacturers plan expanded access programs that include collection of meaningful data outside the highly controlled clinical trial setting, allowing all stakeholders more mechanisms to measure efficacy within the larger patient population. Risks associated with an increase in accelerated access to a broader patient population will be balanced by the knowledge acquired via standardized post-approval data collection. The continuous involvement of the manufacturers and our health regulatory bodies will also distribute responsibility for ongoing monitoring of the patients and drugs beyond clinicians and their respective institutions. Early acquisition of data on a broader cohort of patients will ultimately aid in reimbursement.

Third, we call on all Health Canada, CADTH, and pCPA to create an early intake review panel to consult with manufacturers early in the drug development process. Panel members should include representation from each regulatory body and disease-specific expert clinicians. Consultations and discussions should be designed to help inform the type of data that might aid and expedite health technology reviews and reimbursement decisions. While this results in added regulatory processing costs for manufacturers, especially if the drug does not eventually proceed to the full review process, costs for early panel review discussions might then be subtracted from future fees associated with CADTH and Health Canada submissions should the drug receive priority review. Proactive discussion on the type of data required to approve and ensure access for more of the targeted patient population should allow manufacturers time to implement programs outside of clinical trials to collect such data, including but not limited to expanded access programs, or a pragmatic arm of an ongoing clinical trial. The authors recognize that this recommendation may not be manageable in all countries and might be more realistically feasible if the process is done with global collaboration and co-ordination and incorporated across multiple jurisdictions.

Fourth, we recommend mandating meaningful data collection of all drugs provided outside of clinical trials, including for all SAP approvals and EAP or compassionate use programs. While “meaningful” can be subjective, and may be different for different drugs, guidance from CADTH on RWE and RWD will be helpful to inform data collection. Consultation with physicians, patients, and other collaborative partners about what constitutes meaningful data is also recommended. The program can be modeled on France’s “autorisation temporaire d’utilisation” (ATU) program, which lays out data collection obligations to manufacturers providing their drugs via EAP. Caution should be taken to ensure the added administrative burden placed on clinicians and specialized centers does not become a barrier, and ways to mitigate those concerns should be considered, including decentralizing data collection and allowing outpatient clinics to facilitate and gather data on clinicians’ behalf. Costs associated with such a program should be borne equally among jurisdictions and manufacturers. Transparency must exist regarding the methods of collection, storage, and utilization of these patient data, similar to stringent privacy and confidentiality protections of clinical trials. Analogous, if not identical, protections must be developed for RWD collection. A centralized data tracking method with oversight from Health Canada and CADTH would relieve the potential bias in the interpretation of data collected and analyzed by manufacturers themselves.

Finally, we call on CADTH and pCPA to incorporate more outcome-based reimbursement agreements when there are questions regarding efficacy of rare disease drugs within a given subset of a patient population, rather than immediately preventing its use in some patients through stringent access criteria. Outcome-based agreements can harness RWE and RWD gathered outside of clinical trials. Such agreements should allow access to potentially lifesaving medications for patients while protecting health systems from spending vast amounts on drugs where efficacy is still in question. Outcome-based decision-making will also aid in earlier creation of meaningful start–stop criteria to support decision-making of clinicians, regulatory bodies, and manufacturers. This collaborative start–stop data should be constantly applied and evolving in response to RWD acquisition which will ultimately ensure the drug is being utilized and, from a resource allocation perspective, reimbursed for patients who will derive the most benefit.

## Limitations of this review

While we have tried to be as thorough as possible outlining the many challenges and opportunities in the development and approval of novel therapeutics, the topic is very complex and not all points can be covered and discussed at length. For instance, word count limitations prevent us from commenting and proposing solutions to the extremely high, and oftentimes unjustified, prices of novel therapeutics. For the purposes of our article, we feel that discussion, while salient, detracts from the other substantial issues the regulatory systems have when dealing with the rapidly evolving landscape of orphan drugs. Furthermore, we believe our recommendations can help with the impact these high prices have on health systems by ensuring more data are available to decision makers regarding efficacy for different patient populations, thereby better informing them on which drugs they should, or should not, be reimbursing. In addition, we briefly touched upon the responsibility manufacturers have to ensure novel therapeutics are accessible to patients. However, we have not focused our work on a critique of the pharmaceutical sector and instead kept our priorities centered on overt problems experienced by most health systems and on promoting tangible solutions. Finally, as mentioned above, our article focuses heavily on the details of the Canadian regulatory system. Again, we felt detail was necessary for the sake of clarity. While Canadian centered, we have done our best to highlight the challenges that are common to many health systems and promote recommendations that can be modified and implemented across various jurisdictions.

## Conclusion

The unique nature of novel drugs for the treatment of rare diseases poses challenges in balancing efficiency, cost, fairness, safety, and acquisition of knowledge. The desire for improving access creates several ethical considerations when we deviate from our standard regulatory processes. This requires timely approval of drugs while still preserving ethical principles and methodological rigor in the way we conduct research and monitoring. This warrants a different approach to their study, approval, distribution, and oversight compared to our standard frameworks for other therapeutics.

Here we proposed several changes to the Canadian orphan drug approval and monitoring framework which focus on earlier collaboration and more standardized RWD acquisition as key pillars for change. Mandating a triple-track review process and meaningful data collection ought to be part of this different approach, alongside published CADTH guidance on RWE and RWD. RWD collection must be undertaken in a standardized method which considers the rights and privacies of patients as well as responsibilities placed on clinicians and institutions. In addition, establishing an early intake review panel to collaborate with industry on the type of data that may aid and expedite health technology reviews and reimbursement discussions should proactively mitigate efficacy concerns that have prevented some precision medicines from being accessed by a broader pool of patients. Finally, incorporation of more outcome-based reimbursement agreements and evaluation of appropriate and evolving start–stop criteria should help precision medicines be more accessible to patients in dire need while still protecting health systems from high costs for drugs where efficacy is in question.

Taken together, these suggestions aim to improve the efficiency of accelerated access for patients to novel disease-modifying therapeutics while balancing the challenges arising from accelerated drug approval.

## Declarations

**Ethics approval and consent to participate:** Not applicable.

**Consent for publication:** Not applicable.

**Availability of data and materials:** Not applicable
